# Differential Gene Expression in Rheumatoid Arthritis: Implication in the Diagnosis and Individualized Treatment Plan

**DOI:** 10.26502/jbb.2642-91280187

**Published:** 2025-05-28

**Authors:** Sumanjali Reddy Kanmantha Reddy, Stefanie Au, Ananta Srivastava, Emmanuel Katsaros, Devendra K. Agrawal

**Affiliations:** 1 Departments of Translational Research and Internal Medicine, Western University of Health Sciences, Pomona, California 91766 USA; 2 College of Osteopathic Medicine of the Pacific, Western University of Health Sciences, Pomona, California 91766 USA

**Keywords:** Autoantibodies, Differential gene expression, Disease-modifying antirheumatic drugs, HLA-DRB1 alleles, Inflammation, Methotrexate, Rheumatoid arthritis, Single nucleotide polymorphism

## Abstract

Rheumatoid arthritis (RA) is a chronic autoimmune disease characterized by inflammation and destruction of the joints due to the involvement of biologic, environmental, and genetic factors. Due to its pathogenesis being multifactorial in origin, the underlying molecular mechanisms contributing to the development of RA remain unclear. Therefore, understanding the factors driving RA is crucial for developing targeted therapies and improving patient outcomes. With various genetic variants contributing to RA, this article explores the role of differential gene expression in patients with RA and in different ethnic populations and how the genes contribute to RA susceptibility. Key takeaways from this review demonstrate how HLA shared epitope alleles and non-HLA genes have a strong association with RA and play an important role in immune regulation, autoantibody production, cytokine production, and development of extra-articular manifestations observed in RA. Additionally, gene expression in RA can vary across different sexes and ethnic populations, emphasizing the importance of developing personalized therapeutic interventions. These findings provide insight into the role of differential gene expression in improving diagnostic and therapeutic strategies and highlights potential therapeutic targets for RA management. Future research is needed to determine the clinical relevance of differential gene expression in developing interventions for RA treatment.

## Introduction

Rheumatoid arthritis (RA) is a chronic systemic autoimmune disease that primarily targets the joints but also affects various extra-articular organs, including the skin, eyes, lungs, heart, and kidneys [1]. It is the most common inflammatory arthritis, typically manifesting as symmetric, polyarticular joint pain and swelling, often in the small joints of the hands and feet. However, RA is more than a joint disease, as it presents a continuum of immune dysfunction that progresses over years before becoming clinically apparent [2]. The diagnosis of RA relies on having at least one explained swollen joint, and the more joints that are involved, the more likely patients will be diagnosed with RA. Moreover, confirmatory tests include identifying hallmark features such as persistent joint swelling, elevated inflammatory markers (C-reactive protein and erythrocyte sedimentation rate), and specific antibodies like rheumatoid factor and anti-citrullinated protein antibodies [3]. Imaging and clinical assessments can further confirm the diagnosis and help exclude alternative explanations. Advances in understanding the genetic and environmental contributors to RA have refined the diagnostic process, allowing for earlier detection and treatment. Early and accurate diagnosis is essential for mitigating the risk of systemic complications and improving long-term outcomes. Genome-wide association studies (GWAS) and meta-analyses have identified numerous genetic variants contributing to RA susceptibility, with a significant focus on HLA class II genes, particularly HLA-DRB1 alleles [4]. Specific variants, such as HLA- DRB1*04 and *10, are strongly associated with autoantibody-positive RA, while HLA- DRB1*13 alleles seem to confer protection. Although over 150 candidate loci have been linked to RA, primarily in seropositive cases, the underlying mechanisms remain under investigation, including gene-gene and gene-environment interactions. Advances in pharmacogenomics aim to leverage these genetic insights to personalize treatment strategies, improving patient outcomes and reducing healthcare costs [5]. Ongoing research into diverse populations is expected to uncover additional genetic factors and refine understanding of RA pathogenesis.

## Method

To identify relevant research findings for this literature review on differential gene expression in RA patients, an extensive search was conducted across multiple databases including PubMed, ScienceDirect, and Google Scholar, enabling the selection of high- quality research articles. Key search terms included: “differential gene expression in rheumatoid arthritis”, “SNP”, “immunity”, “autoantibodies”, “inflammatory pathways”, “cytokines”, “DMARD”, “methotrexate”, and “HLA-DRB1”. The search process excluded articles that were not in English. We did not use the terms review, systematic review, meta-analysis, and editorial, and accordingly did not gather information from such articles. PubMed filters were used to ensure that only studies published in English from 2005– 2025 were included. Articles were manually reviewed and commentaries, bibliometric studies, letters to the editor, and papers irrelevant to the research question were excluded. Data regarding the article title and year published were extracted and organized via spreadsheet. Additionally, the content of each article was critically analyzed for the levels of evidence, methods, and key findings.

### Prevalence and Incidence of Rheumatoid Arthritis

**Figure 1:**
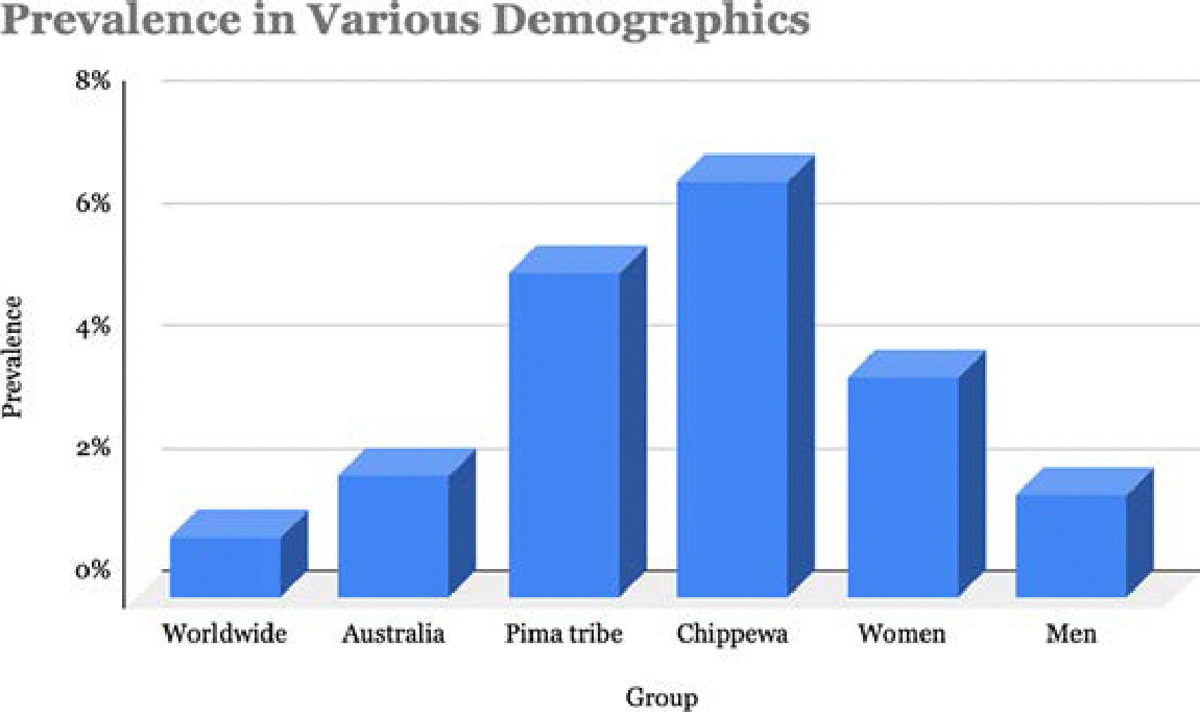
Prevalence of Rheumatoid Arthritis. Worldwide prevalence versus higher prevalence in certain demographics, doubling in Australia, and up to 6.8-fold in Native American tribes such as the Chippewa.

RA is an autoimmune disease that affects approximately 1% of the population worldwide, with its prevalence and incidence varying across different countries and regions [6]. From a global standpoint, the disease is more common in the United States and Europe, and less common in Africa and Asia. However, Australia stands out with the highest prevalence, at approximately 2% [7]. In the United States and western nations of northern Europe, the annual incidence of RA is estimated to be 40 per 100,000 persons [8]. At the community level, RA has the highest prevalence in Native American populations, specifically the Pima (5.3%) and Chippewa (6.8%) tribes (Figure 1), whereas rural populations in South Africa and Nigeria have a very low prevalence of 0.0026% and 0% respectively [7]. Sex is also a strong risk for RA, as it is more prevalent in women compared to men, approximately a 3:1 ratio, and is thought to be a factor that determines disease and symptom severity [9]. The risk of developing RA over a lifetime is 3.6% in women and 1.7% in men [10]. There is also genetic disposition towards RA, as having a first-degree relative with RA increases the risk of developing RA by three-fold [8]. Additionally, the heritability of RA is estimated to be 40–65% for seropositive RA and 20% for seronegative RA, implicating the strong role of genetics in RA pathogenesis [8]. Overall, these variations in prevalence and incidence of RA across different geographical regions suggest the possible influence of various factors contributing to the development of this disease. Therefore, understanding these differences and the underlying factors can allow us to improve the diagnosis and management of RA on a global scale.

### Pathophysiology of Rheumatoid Arthritis

**Figure 2:**
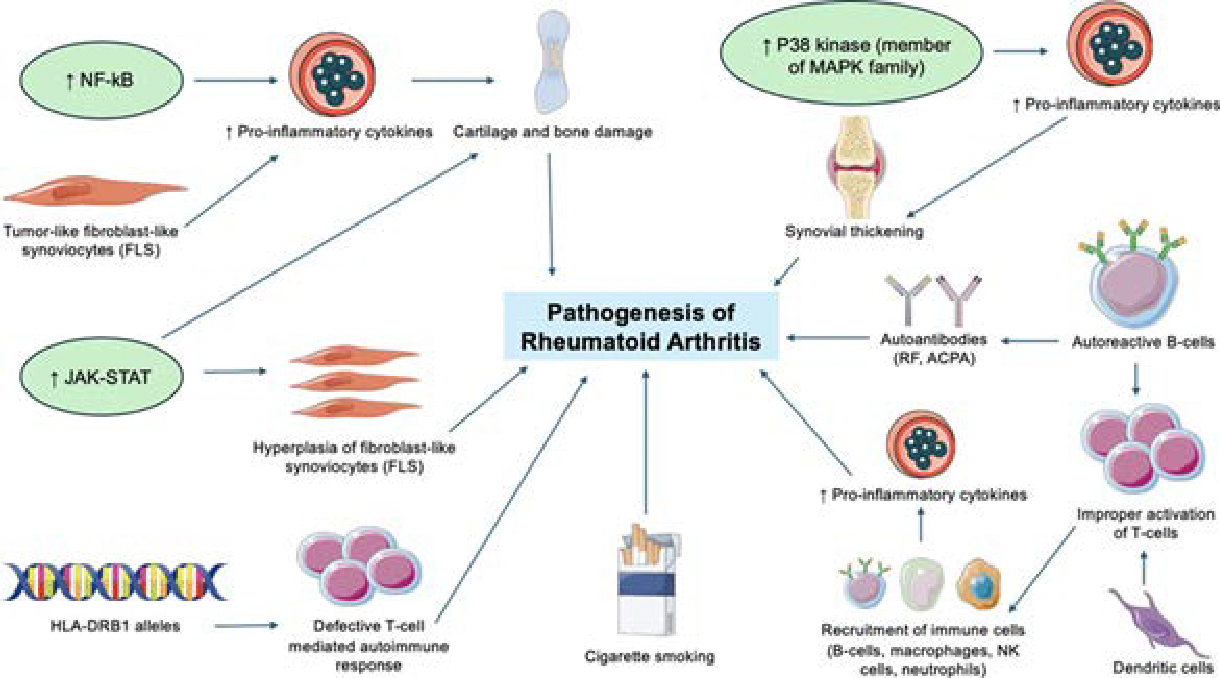
Overview of Underlying Mechanisms of Rheumatoid Arthritis. This illustration demonstrates the various mechanisms contributing to the pathogenesis of RA, including signaling pathways (NF-kB, JAK-STAT, MAPK), fibroblast-like synoviocytes, innate and adaptive immunity, autoantibodies, cytokines, genetics, and environmental factors.

RA is a systemic, chronic inflammatory disease that is complex and multifactorial in origin due to the interplay of biologic, genetic, and environmental factors. RA commonly affects the small joints with the primary manifestation of the disease being symmetrical arthritis of multiple joints such as the hands, feet, wrists, knees [11]. While RA primarily affects the joints, it also has extra-articular manifestations, including, but not limited to, cardiovascular disease, myocardial infarction, pulmonary complications, glomerulonephritis, and keratoconjunctivitis sicca [12,13]. Further, there can also be skin manifestations, especially in severe forms of the disease, such as rheumatoid nodulosis, pyoderma gangrenosum, interstitial granulomatous dermatitis, and etc [14]. The general pathogenesis of the disease involves the body’s immune system attacking the joints, causing inflammation and thickening of the joint capsule, known as synovitis, eventually leading to cartilage and bone damage [11]. Recent studies suggest that a variety of factors including signaling pathways (NF-kB, JAK-STAT, MAPK), fibroblast-like synoviocytes, innate and adaptive immunity, autoantibodies, cytokines, genetics, and environmental factors are involved in the development of RA (Figure 2).

Nuclear Factor Kappa-B (NF-kB) is one of the main inflammatory pathways involved in the pathogenesis of RA as it can induce increased levels of pro-inflammatory cytokines such as IL-1, TNF-ɑ, and IL-6, leading to bone erosion. NF-kB plays an important role in maintaining immune response homeostasis and regulating the cell cycle [15]. However, in RA patients, NF-kB is significantly increased, causing upregulation of pro-inflammatory cytokines, which accelerate the development of RA [11]. In a study investigating the presence of NF-kB in synovial tissue of 13 RA patients and 4 control patients via immunohistochemistry, NF-kB was detected in CD14+ cells within the synovial lining of RA patients, however there was no staining in the synovium of the control subjects [16]. Fibroblast-like synoviocytes (FLS) are cells in the synovium which normally play a role in maintaining synovium homeostasis through building synovial lining, secreting synovial fluid, and lubricating joint proteins. In the setting of RA inflammation, FLS are transformed into tumor-like cells that produce abnormal amounts of inflammatory cytokines and are resistant to apoptosis. However, overactivation of NF-kB can trigger apoptosis of abnormal FLS, leading to accumulation and adhesion of cell debris in joint tissues, causing cartilage and bone damage [11]. The Janus activated kinase-signal transduction and activator of transcription (JAK-STAT) is a key pathway involved in cytokine signaling and plays a crucial role in regulating inflammation. In RA, dysregulation and aberrant activation of the JAK-STAT pathway drives hyperplasia of FLS, synovial inflammation, and degradation of cartilage and bone. The mitogen-activated protein kinase (MAPK) pathway has a variety of functions including regulation of gene expression, cell cycle, apoptosis, etc. Overactivation of MAPKs has been correlated with inflammation of synovial tissues and destruction of articular cartilage. P38 kinase, a key member of the MAPK family, is highly expressed and closely linked to the inflammatory processes seen in RA. When p38 is activated in immune cells such as neutrophils and macrophages, it translocates to the nucleus where it phosphorylates and activates other protein kinases. This leads to the generation of pro-inflammatory cytokines and chemokines, resulting in synovial thickening [11]. The complex interplay of multiple signaling pathways in the pathogenesis of RA highlights their potential as valuable targets for therapeutic intervention (Figure 2).

The interaction between the innate and adaptive immune systems is another factor thought to contribute to the development of RA (Figure 2). The proximity of dendritic cells and T-cells in the synovium suggests the involvement of the adaptive immune system in RA pathogenesis. Dendritic cells are antigen-presenting cells (APCs) that process and present antigens to T cells, playing a role in the first of two signals involved in activation of T cells. The second signal involves interaction between CD80/86 on the dendritic cell with CD28 protein on the T cell. The effectiveness of RA treatment targeting and blocking the interaction involved in the second signal emphasizes the pivotal role of T cells in the pathogenesis of RA [6]. The role of humoral adaptive immunity in RA pathogenesis is highlighted through the role of B cells. Autoreactive B-cells due to defects in B cell tolerance checkpoints can lead to improper activation of T cells and production of pro- inflammatory cytokines, which can contribute to RA pathogenesis. The role of B cells in RA is supported by the successful treatment of RA with Rituximab, a monoclonal antibody that targets the CD20 protein present on B cells shown to alleviate RA symptoms [6,17]. B-cells also play a role in producing autoantibodies that are detected in the serum of RA patients, specifically rheumatoid factor (RF) and anti-citrullinated protein antibodies (ACPA) [17,18]. Rheumatoid factor is an autoantibody against the Fc portion of IgG, while ACPA is directed against citrullinated proteins present in the synovial fluid of RA joints. While RF was the first autoantibody to be detected, it was later discovered that ACPA was more specific for RA [18]. It is postulated that both genetic and environmental risk factors play a role in the formation of these autoantibodies, which can stimulate the release of pro-inflammatory cytokines, such as TNF-ɑ and IL-6, and cause T-cell activation [17,18]. The joint swelling and synovitis present in RA is due to the presence of innate and adaptive immune cells in the synovial compartment. When dendritic cells activate T cells, this leads to recruitment of immune cells such as B cells, macrophages, natural killer cells, and neutrophils into the synovial compartment where they secrete proinflammatory cytokines and matrix metalloproteinases (MMPs) that destruct bone and joints [6,11]. In a healthy individual, the actions of the innate and adaptive immune system are terminated once a pathogen is eliminated, however in the setting of RA, these systems remain chronically activated, driving the chronic inflammation [6]. Thus, the chronic inflammation present in RA is likely driven by a combination of both innate and adaptive immunity mechanisms. Cytokines play a key role in development of inflammation and destruction of joints in RA. This is mainly due to an imbalance between proinflammatory and anti-inflammatory cytokines, such that there is a predominance of proinflammatory cytokines, such as TNF- α, IL-1, and IL-6. TNF-α is produced by activated macrophages, leading to stimulation of B cells, T cells, and natural killer cells while also further inducing the production of more proinflammatory cytokines. This eventually leads to joint destruction and inflammation [17]. The IL-1 family includes IL-1α, IL-1β, IL-18, and IL-33. High concentrations of IL-1β have been found in the plasma and synovial fluid of RA patients and contributes to the characteristic morning stiffness observed in RA [17]. IL-1 can also led to migration of inflammatory cells into the joints and synovium, induce cytokines such as IL-6 and TNF- α, and produce proteoglycans and proteases that lead to pannus formation, which all eventually lead to joint destruction and cartilage damage [19]. IL-6 is another proinflammatory cytokine that is present at elevated levels in the plasma and synovial fluid of RA patients and involved in acute inflammation [19,20]. In RA, IL-6 acts on neutrophils via IL-6R, stimulating the release of proteolytic enzymes and reactive oxygen intermediates while also acting on hematopoietic stem cells to recruit osteoclasts, leading to inflammation, joint destruction, and erosion [21]. The involvement of cytokines in the pathogenesis of RA makes them useful targets for therapeutic interventions. Additionally, cytokine levels can serve as biomarkers to measure disease activity and monitor response to treatment in RA patients.

The interplay of genetic and environmental factors plays a key role in the development of RA. HLA-DRB1 is the most widely studied gene that has the strongest association to RA and is considered a high-risk allele. It is hypothesized that HLA-DRB1 alleles that code a “shared epitope”, a conserved sequence of five amino acids, have a strong correlation with the pathogenesis of RA, resulting in improper presentation of antigens via APCs to T cells, leading to a defective T-cell mediated autoimmune response [11]. Regarding environmental factors, cigarette smoking has the strongest association with the pathogenesis of RA [11,22]. More specifically, smoking is responsible for approximately 20–30% of environmental risk factors associated with RA [23]. It has been found that there is an increased risk of developing RA in HLA-DRB1 individuals who smoke cigarettes [24]. Additionally, in a study that analyzed the effect of smoking on the level of seropositivity for rheumatoid factor and ACPA in RA patients, it was found that smoker patients had higher levels of seropositivity compared to patients who never smoked [25]. In genetically susceptible patients, it has been postulated that smoking leads to increased citrullination, leading to the development of ACPA. Furthermore, smoking has also been associated with the presence of RF independent of RA [23]. Overall, the range of factors involved in RA pathogenesis emphasizes their potential for developing targeted therapies and their role in assessing disease activity.

### Methotrexate and Current Therapies

**Figure 3:**
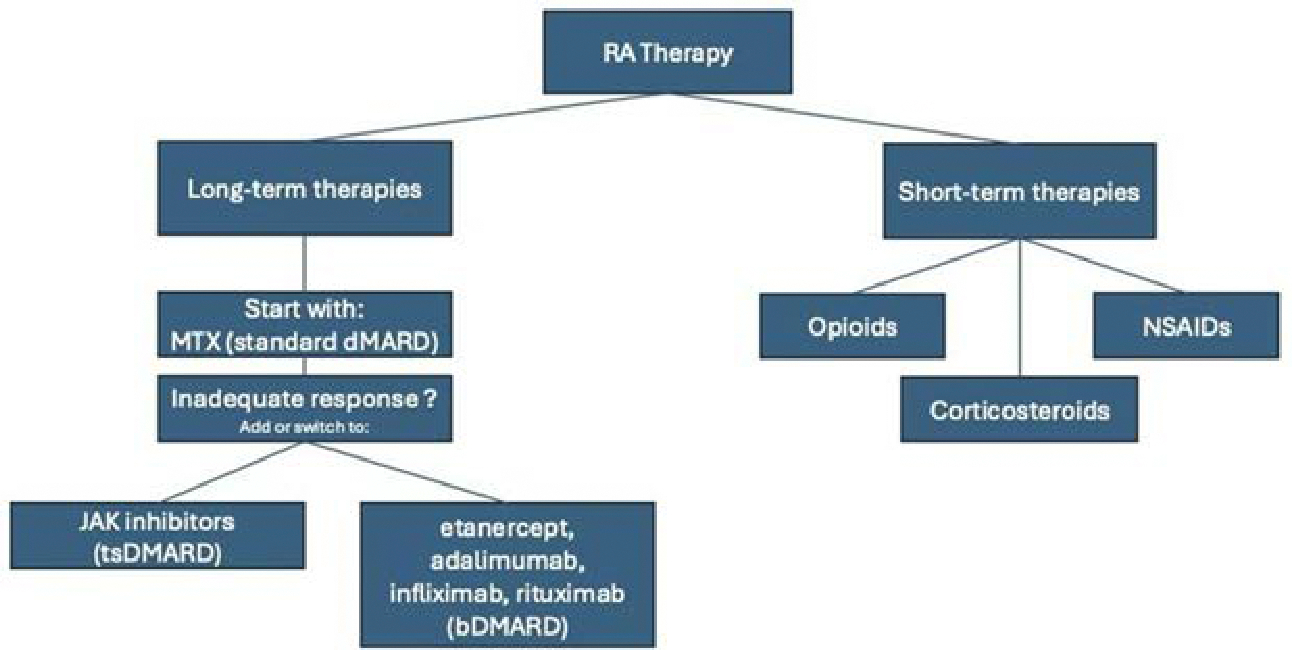
Therapeutics for Rheumatoid Arthritis. This flow chart provides an overview of selected short-term and long-term thematic options for treatment of rheumatoid arthritis.

The primary goal of RA treatment is to mitigate joint destruction, preserve function, and prevent long-term disability (Figure 3). The American College of Rheumatology recommends initiating disease-modifying antirheumatic drugs (DMARDs) within three months of diagnosis to slow disease progression. Methotrexate remains the gold standard DMARD [26,27], serving as the benchmark against which new therapies are compared. Fast-acting medications such as nonsteroidal anti-inflammatory drugs (NSAIDs)— including aspirin, naproxen, ibuprofen, and etodolac—are effective for symptom management but do not alter disease progression [28]. High-dose aspirin, while effective as an anti-inflammatory, has largely been replaced by other NSAIDs due to its side-effect profile. Corticosteroids are more potent anti-inflammatory agents but are reserved for short-term use during disease flares due to their significant side effects [28]. For pain management, weak opioids like codeine, dextropropoxyphene, and tramadol may be used briefly, though their adverse effects often outweigh their benefits [29]. In a 2008 systematic review, it appears that if patients do not show responsiveness to monotherapies like methotrexate, combination therapy has shown improvement, though evidence remains insufficient to recommend a single superior combination strategy for early RA [30]. However, in 2023, real-world data from the CorEvitas RA Registry suggest no significant differences in clinical effectiveness or treatment persistence among biologic and targeted synthetic DMARDs (b/tsDMARD)-naïve patients initiating these therapies, whether used alone or in combination with conventional synthetic DMARDs (csDMARDs) [31].

Targeted therapies such as b/tsDMARDs, including etanercept (ETN), adalimumab (ADA), and Janus kinase inhibitors (JAKi), offer options for patients with an inadequate response to csDMARDs [32]. Biosimilars of adalimumab, infliximab, and etanercept have demonstrated clinically equivalent treatment effects to their reference biologics, offering cost-effective alternatives without compromising efficacy [33]. Combination therapies are another option for patients with insufficient responses to b/tsDMARDs or csDMARDs, tofacitinib, a JAKi, and other biologics, have shown promise both as monotherapy and in combination with MTX [34,35]. Anti-cancer B-cell drugs like rituximab have also shown promise, decreasing the quantity of inflammatory cytokine IL- 40 in RA [36]. Additionally, the clinical implications of antidrug antibodies, which have been demonstrated to affect response in treatments like adalimumab, remain an area of ongoing investigation [37]. Continued research is essential to refine personalized treatment strategies that balance efficacy and safety across diverse patient populations, which is why there are many articles in recent years that have proposed different approaches to treating RA. Recent developments have shown that utilizing mesenchymal stem cell therapy [38], increasing physical activity [39,40], and many other modalities have shown largely positive treatment outcomes, suggesting more trials need to be done to demonstrate their efficacy and safety treating more widespread patient populations.

### Differential Gene Expression in RA Patients

There are a variety of genes that are either upregulated or downregulated in RA, with some being inactive or active, all playing a role in its pathogenesis (Table 1). RA patients produce autoantibodies, which appear to have a genetic association with HLA- DRB1 shared epitope alleles and non-HLA alleles. Genetic analyses have shown that HLA genetic variants have the strongest association with RA, with HLA-DRB1 alleles being considered high risk for autoantibody-positive RA [41]. In a study investigating the association between HLA-DRB1 alleles and susceptibility to RA and production of autoantibodies (ACPA and RF) among RA patients and controls, there was an increased frequency of HLA-DRB1 shared epitope alleles in patients with ACPA-positive RA [42]. More specifically, HLA-DRB1*04 and HLA-DRB1*10 alleles with amino acid valine at position 11 of the HLA-DRB1 gene were found to be high risk for ACPA-positive RA. However, HLA-DRB1*13 alleles with glutamic acid at position 71 of the gene were found to be protective against RA [41]. In a study that analyzed seropositive and seronegative RA in association with various HLA alleles, it was found that amino acids leucine and serine at position 11 of HLA-DRB1 were considered high risk for ACPA-negative RA [43,44]. The HLA-DRB1 shared epitope alleles have also been found to have a gene dosage effect, such that there is increased disease severity as the number of these alleles increase. Furthermore, these alleles have also been associated with a greater risk of developing extra-articular manifestations of the disease. With HLA-DRB1 alleles contributing to approximately 30% of the genetic component of RA, it is evident that HLA variants have a significant genetic association with the development of RA [45,46,47]. While HLA shared epitope alleles have the strongest association with RA, there are also non-HLA genes, such as PTPN22 and IL2 receptor gene, associated with RA (Table 1). In a genome wide association study conducted in 2007 by the Wellcome Trust Case- Control Consortium (WTCCC), it was found that the 1858 C/T polymorphism of the PTPN22 gene was significantly associated with RA. The protein produced by PTPN22 appears to have a role in reducing the responsiveness of T and B cell receptors, which appears to be diminished with the polymorphism present. This study also found that a single nucleotide polymorphism (SNP) of the alpha and beta regions of the IL-2 receptor, specifically IL2RA and IL2RB, had moderate association with RA due to the role of IL-2 receptor in regulating the stimulation of T cells via IL2 [45]. Additionally, according to a variety of GWAS, SNPs in PAD14, STAT4, and CTLA4 were all also associated with RA susceptibility. The PAD14 gene produces PAD14, which is an enzyme that plays a role in citrullination of arginine residues during post-translational modification and has an association with the production of ACPA in RA. STAT4, known as signal transducer and activator of transcription 4, is induced by cytokines and regulates differentiation of T- helper cells [48,49]. It was found that this is overexpressed in the synovium of RA patients. CTLA4, a cytotoxic T-lymphocyte associated antigen, plays a role in inhibiting T-cell activation. SNPs associated with this gene lead to a lack of inhibitory function and increase susceptibility to ACPA-positive RA [48].

In a study that investigated Th17/Treg transcriptional factor expression in patients with RA compared to healthy controls, it was found that Treg was lower, but Th17 was higher in RA patients compared to healthy controls. While Th17 induces pro-inflammatory cytokines, Treg has an anti-inflammatory role, involved in suppressing Th17 and inhibiting autoimmunity. There are certain transcriptional factors that characterize Th17, such as STAT3 and STAT5, which contribute to RA pathogenicity. STAT3 is crucial for Th17 differentiation while STAT5 blocks Th17 differentiation, however in RA patients, no expression of STAT5 and elevated expression of STAT3 were observed in Th17 cells, leading to enhanced pro-inflammatory activity Th17 due to a lack of inhibition of Th17 differentiation [50]. In another study, long intergenic non-protein coding (lnc) RNA 00305 (LINC00305), a pro-inflammatory lncRNA associated with atherosclerosis, was found to be significantly increased in RA patients and associated with elevated C-reactive protein, erythrocyte sedimentation rate, rheumatoid factor, and ACPA. Furthermore, it was found that the rs2850711 polymorphism of LINC00305 was significantly associated with an increased risk of RA [51]. As discussed in the RA pathophysiology section above, matrix metalloproteinases (MMPs), produced by RA synovial fibroblasts, play a key role in the pathogenesis of RA and are thought to be involved in epigenetic mechanisms that upregulate the MMP gene. Epigenetic mechanisms such as DNA methylation, histone modifications, and disordered microRNA (miRNA) expression have been shown to cause abnormal MMP gene expression and activation, contributing to the characteristic cartilage destruction seen in inflamed joints during RA pathogenesis [52]. Lastly, the potential role of recombination signal-binding protein for immunoglobulin kappa J region (RBPJ), a transcriptional regulator involved in the modulation of immune homeostasis and cellular differentiation, was examined in a study. When the expression of RBJP among RA patients and healthy controls was quantified, it was found that RBPJ was expressed at lower levels in RA patients compared to healthy controls. In animal studies, it was found that RBJP may be involved in T cell differentiation and signaling, further supporting its potential role in RA [53].

Analysis of gene expression in RA patients has also provided insight into sex differences in RA. A study that identified the key genes associated with sex differences in RA found that there were approximately 1169 differentially expressed genes, with 845 upregulated genes and 324 down regulated genes, that overlapped between males and females with RA. Based on maximum correlation criteria, the investigators then used a software tool to identify the top ten genes associated with the development of RA in both males and females. The upregulated genes include UBE2E1, EGF, PIK3R1, UBE2G1, HRAS, PTPN11, and NDUFV1, while the downregulated genes include RAC1, UQCRB, and UBA1. This study also suggested that the MAPK pathway and autophagy may be playing a key role in the sex differences observed in RA [54]. Overall, the genetic variants associated with the pathogenesis of RA play a crucial role in improving our understanding of the disease as well as advancing therapeutic interventions as they have the potential to further our understanding of pharmacogenomics in RA.

### Differential Gene Expression in Different Ethnic Populations

Gene expression in RA can vary among different ethnic populations, highlighting the genetic diversity underlying the disease. As discussed earlier, HLA-DRB1 has a strong genetic link to RA, however it was found that the specific HLA-DRB1 alleles vary among Asian and European populations. While valine is present at position 11 of the HLA-DRB1 gene in European populations corresponding to the HLA-DRB1*04 and HLA- DRB1*10 alleles, in the Asian population, the HLA-DRB1*09 haplotype with aspartic acid instead of valine at position 11 of HLA-DRB1 appeared to be a risk allele for the Asian population [41]. The HLA-DRB1*09 allele was significantly associated with RA in the Japanese population [55]. Furthermore, in Asian populations, there was a strong association of seropositive RA with HLA-DRB1*04:05 allele, which appears to be rare in African and European populations [41,56]. A genome-wide association study that aimed to explore the genetics of RA in the Arab population identified a significant association between RA and amino acid position 11 of the HLA-DRB1 gene, like findings in European and Asian populations, suggesting genetic similarity in RA among certain ethnic groups [57,58]. HLA-DOA is a non-classical HLA gene examined in a study for its possible contribution to ACPA-positive RA risk. This study found that this HLA-DOA variant is most prevalent in the Japanese population compared to other ethnic groups, contributing to RA development in this population [59]. Regarding non-HLA variants, it was found that the PTPN22 polymorphism was significant in the European populations but rare in populations of Asian descent [45,60]. More specifically, the exonic SNP rs2476601 of the PTPN22 gene is rare in East Asian populations [41]. A meta-analysis of PAD14 polymorphisms found that while five PAD14 polymorphisms (PAD14_89, PAD14_90, PAD14_93, PAD14_94, PAD14_104) were significantly associated with RA in the Asian population, only PAD14_94 was associated with RA in the European population [48,61]. In a study that aimed to identify novel genes associated with RA susceptibility with a focus on ethnic differences among European and Asian populations, a total of 221 genes associated with RA were identified, with 71 that overlapped among both populations, 76 that were specific to the European population, and 74 that were specific to the Asian population. However, 105 of these genes had significant differential gene expression among RA patients and healthy controls, with 20 being the most significant. Of these 20 genes, there were 11 genes that overlapped among both populations, 5 genes specific to the European population ((PHTF1, RPS18, BAK1, TNFRSF14, SUOX)), and 4 genes specific to the Asian population (RNASET2, HFE, BTN2A2, MAPK13) [62]. NLPR1 is a gene that has been shown to be a risk factor for some inflammatory and autoimmune diseases such as vitiligo, Addison’s disease, type 1 diabetes mellitus, etc. In a study exploring the potential link between NLPR1 gene polymorphism and RA development in Han Chinese, it was found that the rs878329 polymorphism was associated with RA in this population [63,64,65]. Lastly, it is known that TNF-α is a major cytokine involved in the pathogenesis of RA through the production of more proinflammatory cytokines as discussed earlier. DNA microsatellites are short, repetitive sequences in the non-coding regions (introns) of the genome that can affect DNA folding and transcription rates. A study examining the association between tumor necrosis factor microsatellite alleles and RA susceptibility in Indian patients found that microsatellite TNFβ5 allele was increased in patients compared to healthy controls. Additionally, patients with higher frequency of this allele exhibited increased disease severity, suggesting that the TNFβ5 allele may serve as a risk factor for RA in the Indian population [66,67]. Based on these findings, the variation in gene expression and RA incidence across different ethnic populations [70] highlights the importance of considering ethnic-specific genetic factors into the diagnosis and management of RA to provide individualized and effective care to patients.

## Conclusion

RA is a complex inflammatory disease, and to further understand factors that contribute to its pathogenesis, it is crucial to identify how certain gene expressions may influence the progression of the disease. The literature highlights that specific genetic and epigenetic mechanisms can increase the risk for RA or disease severity while others may provide protective effects. Furthermore, other studies have identified variations across different ethnic groups. Better understanding of the differential gene expressions in RA is essential for advancing our knowledge of the disease and enhancing individualized treatment plans to improve patient outcomes.

## Figures and Tables

**Table 1:** HLA vs. non-HLA Variants in Rheumatoid Arthritis (RA) Pathogenesis. This table summarizes the HLA-DRB1, and non-HLA gene variants associated with RA and their role in RA pathogenesis through their various mechanisms.

HLA vs. non-HLA Variants in RA Pathogenesis	
HLA Variants	
Variant	Role
HLA-DRB1*04 and HLA-DRB1*10 (amino acid valine at position 11 of the HLA- DRB1 gene)	High-risk for ACPA-positive RA
HLA-DRB1*13 (amino acid glutamic acid at position 71 of the HLA-DRB1 gene)	Protective against RA
Amino acids leucine and serine at position 11 of HLA-DRB1	High-risk for ACPA-negative RA
Non-HLA Variants	
Variant	Role
*PTPN22* (1858 C/T polymorphism)	*PTPN22* plays a role in normal functioning of T and B cell receptors
IL2 receptor (SNP of the alpha and beta regions)	IL2 is involved in regulation of T cell stimulation
*PAD14* (SNP)	PAD14 protein plays a role in citrullination of arginine residues in post-translational modification and production of ACPA
*STAT4* (SNP)	*STAT4* regulates T-helper cell differentiation
*CTLA4* (SNP)	*CTLA4* is involved in regulating T-cell activation, specifically its inhibition
Th17/Treg transcriptional factor expression (high Th17 and low Treg)	Th17 induces pro-inflammatory cytokines; Treg has anti-inflammatory properties, including suppressing Th17 and inhibiting autoimmunity
Long intergenic non-protein coding (lnc) RNA 00305 (LINC00305) (increased)	LINC00305 has pro-inflammatory properties and is associated with elevated CRP, ESR, RF, and ACPA rs2850711 polymorphism of LINC00305 associated with increased risk of RA
Matrix metalloproteinase (MMP) (upregulated)	Epigenetic mechanisms upregulate MMP gene expression and activation, leading to cartilage destruction and inflamed joints
Recombination signal-binding protein for immunoglobulin kappa J region (RBPJ) (low levels)	RBPJ modulates immune homeostasis and cellular differentiation, specifically T- cell differentiation and signaling

## Key points, Outstanding Questions, and Challenges

- HLA shared epitope alleles and non-HLA genes have a strong association with Rheumatoid Arthritis (RA) and play a crucial role in various aspects of the disease such as severity and greater risk of extra-articular manifestations.
- Gene expression in RA can vary across different sexes and ethnic populations, highlighting the need for personalized therapeutic interventions.
- Differential gene expression provides insights into improving diagnostic and therapeutic strategies and highlights potential therapeutic targets for RA management
- What are the precise molecular mechanisms by which these specific gene variants (e.g., HLA-DRB1 alleles, PTPN22 polymorphism) contribute to the initiation and progression of the disease at the cellular and tissue level?
- How do environmental factors interact with the identified sex- and ethnicity-specific gene expression patterns to influence RA susceptibility and disease course?
- While the literature covers RA in European and some Asian patients, there fails to be adequate research on the rest of the world despite current literature stating RA to be more prevalent in developed areas.
